# Gut microbiota characteristics in mice with antibiotic-associated diarrhea

**DOI:** 10.1186/s12866-020-01999-x

**Published:** 2020-10-15

**Authors:** Haoqing Shao, Chenyang Zhang, Nenqun Xiao, Zhoujin Tan

**Affiliations:** 1 School of Traditional Chinese Medicine, Hunan University of Chinese Medicine, Changsha, Hunan China; 2 Hunan Key Laboratory of TCM Prescription and Syndromes Translational Medicine, Changsha, Hunan China; 3 School of Pharmaceutical Science, Hunan University of Chinese Medicine, Changsha, Hunan China; 4 School of Medicine, Hunan University of Chinese Medicine, Changsha, Hunan China

**Keywords:** Gut microbiota, Antibiotic-associated diarrhea, Gentamicin, Cefradine, *Enterococcus*, *Clostridium*, 16S rRNA gene sequencing

## Abstract

**Background:**

Antibiotic-associated diarrhea (AAD), defined as diarrhea that occurs in association with the administration of antibiotics and without another clear etiology, is one of the most common adverse drug events of antibiotics therapy. We established a diarrhea model induced by gentamycin and cefradine to investigate the microbiota characteristics in the intestinal lumen of mice with AAD and provide insights into noteworthy bacteria related to gentamicin and cefradine-associated diarrhea.

**Results:**

The number of OTUs in the model group and the normal group was 983 and 2107, respectively, and 872 identical OTUs were shared between two groups. Species richness and species diversity of intestinal microbe were altered by antibiotics administration. PCoA showed a clear separation between AAD and health control. The dominant phyla of AAD mice were Firmicutes (52.63%) and Proteobacteria (46.37%). Among the genus with top 20 abundance, the relative abundance of 7 genera, *Ruminococcus*, *Blautia*, *Enterococcus*, *Eubacterium*, *Clostridium*, *Coprococcus*, and *Aerococcus*, were enriched in the model group. Based upon the LEfSe analysis, *Enterococcus*, *Eubacterium*, *Ruminococcus*, and *Blautia* were identified as potential biomarkers for AAD.

**Conclusions:**

The bacterial diversity of the intestinal lumen was diminished after gentamicin and cefradine administration. The alterations in the abundance and composition of gut microbiota further led to the dysfunction of gut microbiota. More specifically, gentamicin and cefradine significantly increased the abundance of the opportunistic pathogens, of which *Enterococcus* and *Clostridium* were the most prominent and most worthy of attention.

## Background

Antibiotics are frequently prescribed drugs for clinical treatment of various bacterial infections. Yet several adverse drug events (ADE) have emerged as the widespread use of antibiotics. Antibiotic-associated diarrhea (AAD), defined as diarrhea that occurs in association with the administration of antibiotics and without another clear etiology, is one of the most common ADE of antibiotics administration [1].

AAD is a complex disease that is affected by the host, infectious agent involved, and numerous clinical elements, including antibiotics therapeutic scheme. AAD can affect up to a third of the patients receiving a particular antibiotic [2], but the incidence and severity of AAD vary among different antibiotics. We observed more serious diarrhea in mice which received cephradine + gentamycin sulfate than in mice which received lincomycin hydrochloride + ampicillin sodium and ceftriaxone sodium + erythromycin lactobionate [3]. Numerous studies reported that the mechanisms for AAD mainly laid on the changes or dysbiosis of microbial composition and function induced by antibiotics [4–7]. Although recent studies were concentrated on *C. difficile*-associated diarrhea, it accounts for only 10–25% of all AAD cases [7]. The etiological factor in the majority of AAD cases remains undetermined. Bacteria such as *Clostridium perfringens*, *Staphylococcus aureus*, and *Klebsiella oxytoca* were also related to AAD [8]. Understanding the different etiological factors and pathogenesis that implicated in AAD may help to prevent AAD and reduce costs.

Gut microbiota is the trillions of microorganisms that normally inhabit the intestine of humans and other animals [9]. It is currently considered to be a highly complex ecosystem in which there are tremendous interdependencies and interactions between microbial species and between the microbes and their host [5]. The gene pool of the microbial inhabitants is considerably diverse and almost 100 times larger than the gene pool of the host [10]. Gut microbes contribute functional genes and metabolites which affect host metabolism, immune, endocrine, and other physiological processes and are, therefore, increasingly recognized as a necessary and key factor for maintaining health [11]. The microbial composition of the gut microbiota can be influenced by lifestyle, diet as well as drugs and varied across the digestive tract. The colon, containing a densely-populated microbial ecosystem with up to 10^12^ cells per gram of intestinal content, harbors much greater microbial abundance and diversity relative to the small intestine [12]. Consequently, microbial analyses of intestinal content or stool have been wildly adopted as measures to surmise the role of gut microbe in host health and disease, based on the belief that it represents all microbial populations throughout the intestines [12]. Numerous studies have confirmed that disruptions in the composition and function of the gut microbe are associated with diseases ranging from localized gastrointestinal disorders to neurological, respiratory, metabolic, hepatic, and cardiovascular illnesses [13–15]. Many efforts are currently focused on exploring potential causality and related microbe-mediated disease mechanisms, with the hope that an improved understanding will fuel the conception and realization of novel therapeutic and preventive strategies [16]. There is increasing evidence for the effectiveness of probiotics, particularly *Lactobacillus rhamnosus* or *Saccharomyces boulardii*, and fecal microbiota transplantation in preventing or treating AAD [17, 18]. Advances in our understanding of microbiota characteristics might set the foundation for designing therapies that target the gut microbiota to prevent and treat AAD.

In the previous study, we found that a combination of gentamycin and cefradine showed a higher diarrhea incidence than a combination of lincomycin and ampicillin and a combination of ceftriaxone and erythromycin [3]. To further investigate potential pathogens responding to gentamicin and cefradine-associated diarrhea and better understand the different mechanisms of AAD, we analyzed the characteristics of gut microbiota in AAD model mice and healthy individuals.

## Results

### Analysis of 16S rRNA gene sequences and operational taxonomic unit

**Fig. 1:**
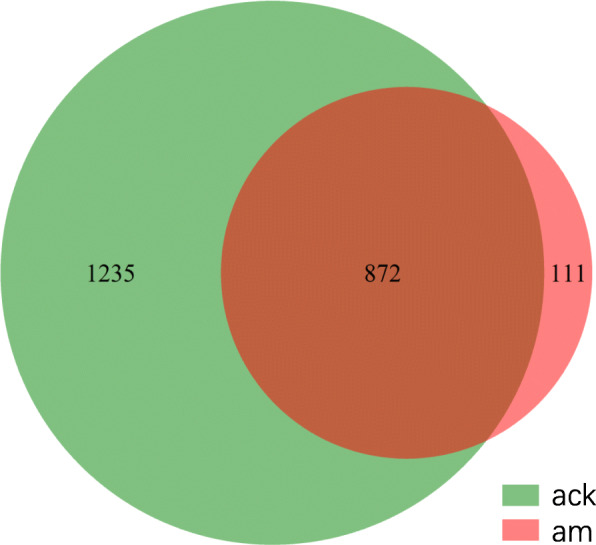
Comparison of OTUs in the two groups by Venn diagram (at distance 0.03). ack: control group, am: model group. The character ‘a’ represents the antibiotic-associated diarrhea experiment, ‘ck’ represents the control, and ‘m’ represents the model

A total of 766,407 high-quality sequences were detected from 6 samples belonging to two groups, with a length distribution concentrated at 225 bp. The Good’s coverages calculated by Mothur to reflect the depth of sequencing were greater than 99.75% (from 0.9975 to 0.9997), which indicates that the sequencing results represent the true condition of the microorganisms in the sample. Finally, these high-quality sequences were clustered into 2218 OTUs (after quality-filtering) based on similarity at a 97% threshold. The number of OTUs in groups control and model was 2107 and 983, respectively (Fig. 1). There were 872 identical OTUs between the two groups, and the percentage of identical OTUs was 39.31%.

### Diversity analysis of intestinal microbiota

**Fig. 2:**
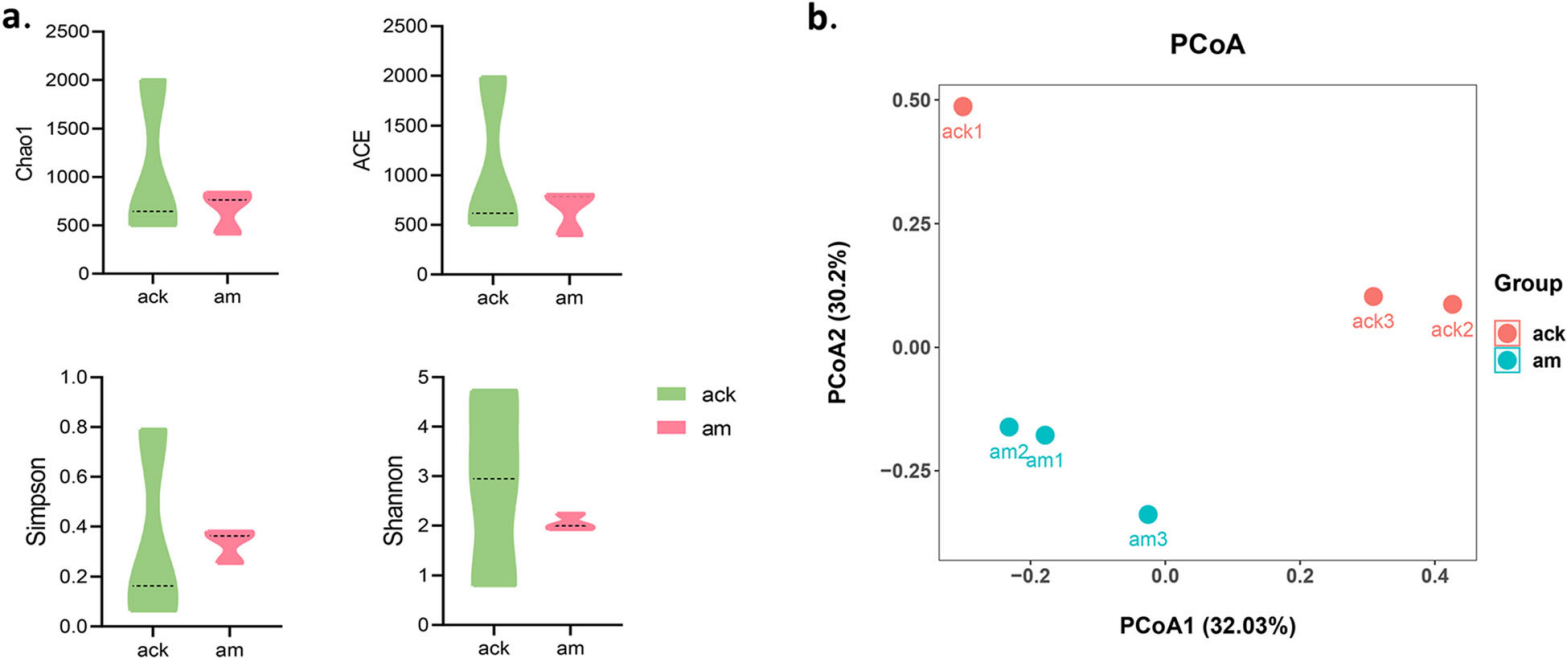
Diversity analysis of gut microbiota. **a** Grouping histogram of Alpha diversity indexes. Data are $\overline{x}$ ± SD, *n* = 3, *p* > 0.05 (Mann-Whitney U test). **b** PCoA plot based on unweighted UniFrac distances. Ack: control group, am: model group

Alpha diversity indexes are regularly adopted in ecology to estimate the richness of microbial species and quantitatively describe microbial species diversity in a community. The greater the Chao1 and ACE index, the richer the species in a community. The larger the Shannon value and the smaller the Simpson value, the higher the diversity in a community. In this study, the Chao1, ACE, Simpson, and Shannon index in the model group were 672.28 ± 227.51, 664.43 ± 232.91, 0.33 ± 0.07, and 2.06 ± 0.17, which were lower than 1049.31 ± 828.04, 1035.84 ± 825.48, 0.34 ± 0.39, and 2.83 ± 1.97 in the control group (Fig. 2a).

Beta diversity measures the change in the diversity of species from one environment to another. The principal coordinates analysis (PCoA) based on unweighted UniFrac distances (accounting for the abundance of OTUs) was performed to explore and to visualize the dissimilarities of microbial communities between two groups (Fig. 2b). The results showed that, on the whole, the distance between groups was further than that within groups meaning that the microbial communities were different between the model group and normal group.

### Comparison of gut microbiota at the phylum level

**Fig. 3:**
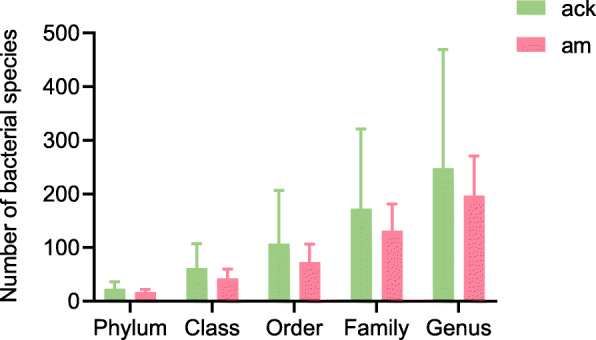
The number of microbial species of each sample at different taxonomic levels. Data are $\overline{x}$ ± SD, *n* = 3, *p* > 0.05 (Mann-Whitney U test). ack: control group, am: model group

**Fig. 4:**
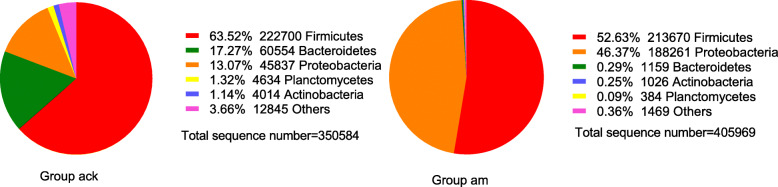
Gut microbiota structure at the phylum level. Ack: control group, am: model group

After OTUs less than 0.001% were filtered, the remaining OTUs were classified into 38 phyla, 115 classes, 223 orders, 347 families, and 516 genera (Fig. 3). Overall, the dominant phyla of the gut microbiome in mice were changed from Firmicutes, Bacteroidetes, and Proteobacteria to Firmicutes and Proteobacteria after antibiotics administration. Compared to the control group, the model group had an over-representation of Proteobacteria (13.07% vs 46.37%)*,* and lower abundances of Firmicutes (63.52% vs 52.63%), Bacteroidetes (17.27% vs 0.29%), Actinobacteria (1.14% vs 0.25%) and Planctomycetes (1.32% vs 0.09%) (Fig. 4). There was a significantly higher Firmicutes to Bacteroidetes (F/B) ratio in samples of AAD model group (*p*FDR < 0.05).

### Antibiotics changed the dominant bacteria of intestinal contents at the genus level

**Fig. 5:**
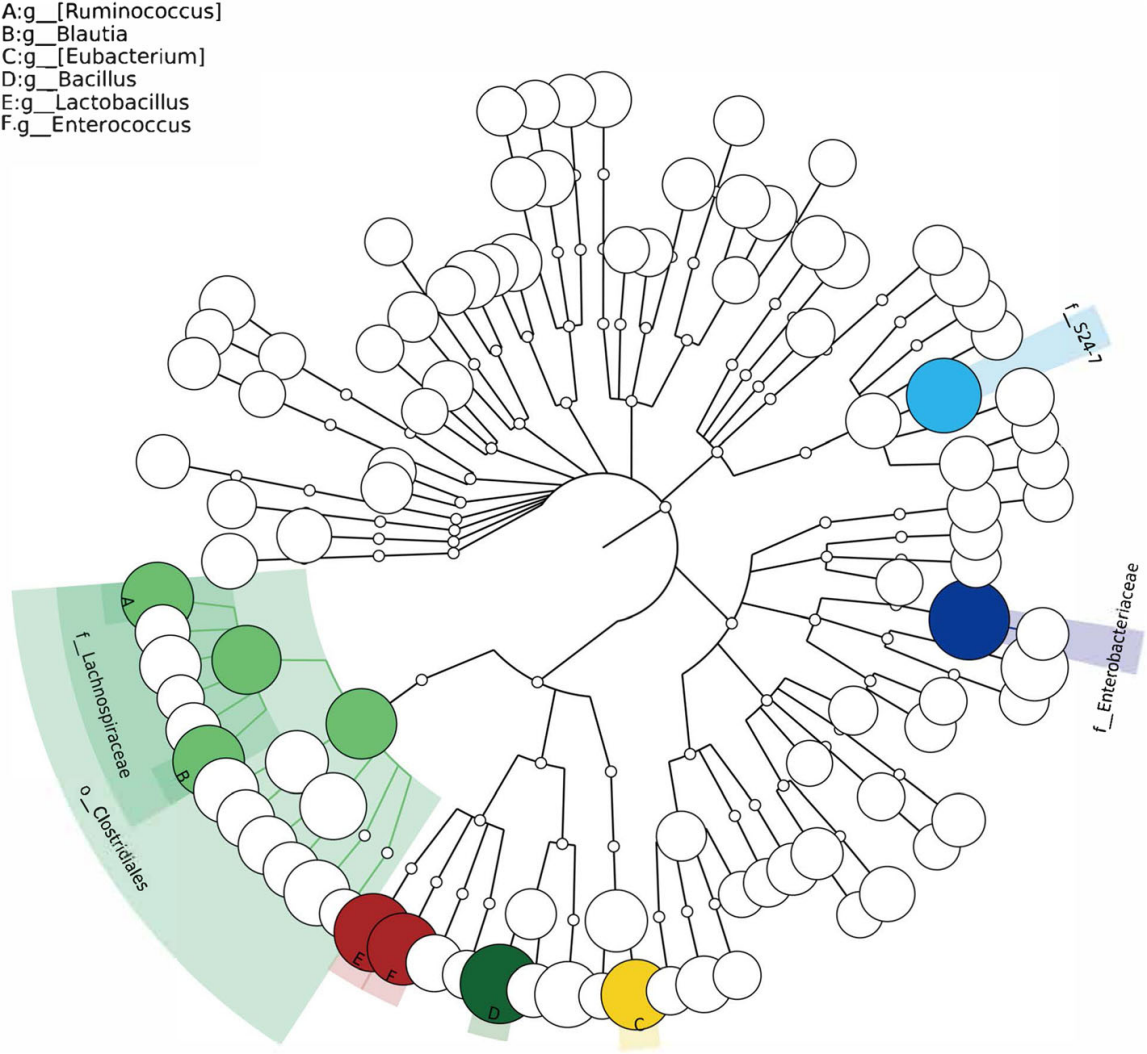
Phylogenetic dendrogram analysis based on species and abundance of mice gut microbe at the genus level. The node size represents the average relative abundance, and species with abundance > 1% were shown in color

**Fig. 6:**
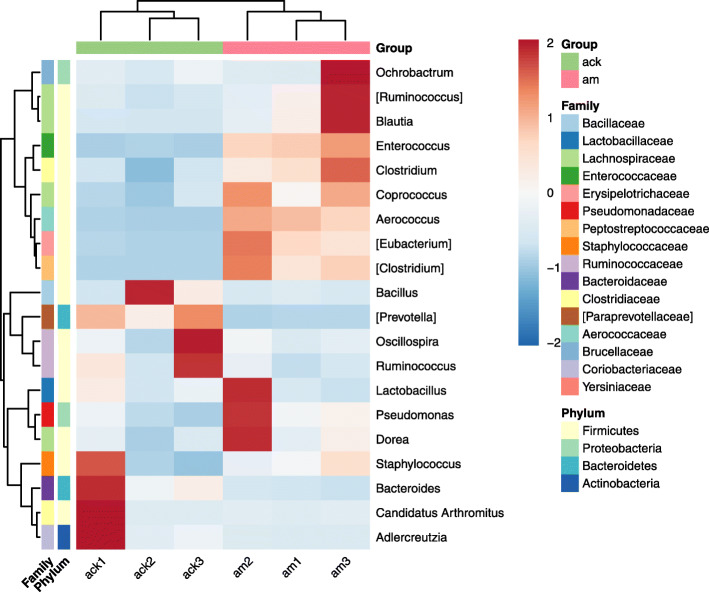
Heat map of top 20 genera in abundance. The darker the red color, the higher the relative abundance is. The darker the blue color, the lower the relative abundance is. ack: control group, am: model group

A phylogenetic dendrogram was performed to reflect the information of species and abundance of mice gut microbiome (Fig. 5). *Bacillus*, *Lactobacillus*, *Ruminococcus*, *Enterococcus*, *Blautia,* and *Eubacterium* were especially showed in color based on their abundance of over 1%. The top 20 genera in abundance were shown in a heat map (Fig. 6). Among the genus with the top 20 abundance, the relative abundance of 7 genera, *Ruminococcus*, *Blautia*, *Enterococcus*, *Eubacterium*, *Clostridium*, *Coprococcus*, and *Aerococcus*, were enriched in the model group. Moreover, genera with abundance over 1% in group model were *Lactobacillus* (23.21%), *Enterococcus* (5.97%), *Blautia* (5.46%), *Ruminococcus* (5.37%), *Bacillus* (5.24%), *Eubacterium* (4.62%), *Pseudomonas* (2.09%) and *Clostridium* (1.79%). However, there are only 2 genera with an abundance of over 1% in group control, which is *Bacillus* (40.46%) and *Lactobacillus* (13.43%).

### Taxa with significantly different abundances between groups by LEfSe

**Fig. 7:**
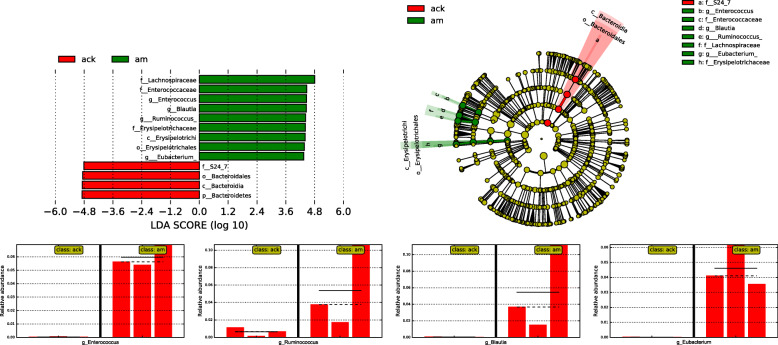
Taxa with significantly different abundances between groups by LEfSe. The horizontal straight line in the panel indicates the group means, and the dotted line indicates the group medians. Ack: control group, am: model group

To determine the variations in gut microbiota composition between the normal and AAD model mice, linear discriminant analysis effect size (LEfSe) was performed to find which taxa were enriched in the two groups (Fig. 7). LEfSe emphasizes both statistical significance and biological relevance, allowing researchers to identify differentially abundant features that are also consistent with biologically meaningful categories (subclasses) [19]. Although LEfSe revealed that the genera *Enterococcus*, *Eubacterium*, *Blautia*, and *Ruminococcus* were the most abundant in the model group, this dominance of the latter two was not observed in all three samples of the model group (Fig. 7). Relatively increased numbers of *Enterococcus* and *Eubacterium* were observed in all three samples of model group, indicating that the two genera were possibly related to the occurrence of AAD in model mice.

## Discussion

Changes in intestinal bacterial composition caused by antibiotics vary from antibiotic to antibiotic. Gentamicin is a broad-spectrum aminoglycoside with strong antibacterial activity against gram-negative bacteria. Cefradine, a broad-spectrum cephalosporin belongs to β-lactam, has a bactericidal effect on both gram-positive and gram-negative bacteria. In the previous study, we found the numbers of bacteria and colibacillus decreased rapidly with the use of gentamicin and cefradine. The number of yeast and mold presented an increasing trend [20]. Similar changes were found in other AAD models. Theriot et al. [21] treated mice with a variety of antibiotics to create distinct microbial and metabolic (bile acid) environments and found that susceptibility to *C. difficile* in the large intestine was observed only after specific broad-spectrum antibiotic treatment (cefoperazone, clindamycin, and vancomycin). These changes were correlated to the loss of members of families Lachnospiraceae and Ruminococcaceae [21]. Larcombe et al. [22] established an *S. aureus* infection model in mice pre-treated with kanamycin, gentamicin, colistin, metronidazole, vancomycin, and cefaclor. The results showed that colonization of various *S. aureus* strains could be achieved after antibiotic pre-treated [22]. Antibiotics lead to an alteration in bacteria composition resulting in changed metabolism and diminished anti-colonization.

The results, as direct evidence, suggested that the mice developed AAD was associated with alteration of the normal gut microbiota, which was mainly manifested as fewer beneficial bacteria and more potential pathogens. As shown in Fig. 3, after treatment with antibiotics, the number of bacterial species in intestinal contents decreased to different degrees at the levels of phylum, class, order, family, and genus. This result indicated that antibiotics changed the structure and density of normal intestinal flora, resulting in the disorder of intestinal flora. Specifically, to the level of phylum, the gut microbiota of normal mice was comprised of three dominant phyla, namely Firmicutes (63.52%), Bacteroidetes (17.27%), and Proteobacteria (13.07%). As for AAD mice, it was transformed into Firmicutes (52.63%) and Proteobacteria (46.37%). There was a significantly higher F/B ratio in AAD mice. Similar shifts occurred in the intestinal mucosal bacteria of AAD mice [23]. Massive data identified Proteobacteria as a possible microbial signature of diseases which are sustained by various degrees of inflammation [24]. Notably, inflammation is demonstrated to be implicated in the development of metabolic disorders [25]. Thus, an increased abundance of Proteobacteria implies the risk of infection and metabolic disorder in a pathological state.

Furthermore, significant alterations in the gut microbiota composition were found at the genus levels. The dominant genera of healthy mice were *Bacillus* and *Lactobacillus*, which were beneficial to maintaining healthy intestinal flora and reducing the colonization of pathogenic organisms [26, 27]. However, with the administration of antibiotics, bacteria that were sensitive to gentamicin and/or cefradine were suppressed or killed, and bacteria that were resistant to them have the opportunity to invade and multiply. As a consequence, *Ruminococcus*, *Blautia*, *Enterococcus*, *Eubacterium*, *Clostridium*, *Coprococcus*, and *Aerococcus* were enriched in AAD mice (Fig. 6). Among these bacteria, the changes of *Enterococcus* and *Eubacterium* were especially prominent (Figs. 6, 7). In the AAD mice, the abundance of *Enterococcus* and *Clostridium* showed a significant increase, which was similar to the changes in the intestinal mucosa of AAD mice [23]. In our previous studies, we found a significant reduction in *Lactobacillus* in the intestinal mucosa of AAD mice, and the main genera in the intestinal mucosa of AAD mice were *Enterococcus*, *Stenotrophomonas*, *Glutamicibacter*, *Citrobacter*, and *Pseudomonas* [23]. The main genera in the intestinal contents of the same AAD model mice were *Lactobacillus*, *Enterococcus*, *Blautia*, *[Ruminococcus]*, and *Bacillus*. Previous studies also showed that the role of intestinal microbiota in the development of AAD from the perspective of intestinal microbial function enzyme (lactase) gene. The main lactase-producing strains differed in the intestinal content and mucosa. The main lactase-producing strain in the intestinal contents is *Pseudomonas fluorescens* [28], while the main lactase-producing strain in the intestinal mucosa is *Stenotrophomonas* [29]. Besides, antibiotics reduced the diversity of bacterial lactase genes in the intestinal contents but increased it in the intestinal mucosa [28, 29]. These dissimilarities in the composition and function of intestinal mucosal microbiota and intestinal contents microbiota provide new evidence for the spatial heterogeneity along the cross-section of the digestive tract (from lumen to mucosa). Factors known to drive this spatial heterogeneity along the longitudinal and transverse axes include chemical gradients (e.g., pH), oxygen levels, nutrient availability, immune effectors, and functional heterogeneity of each gastrointestinal tract segment [12, 30].

*Enterococcus* are important opportunistic pathogens, with *E. faecalis* and *E. faecium* as the most representative species, causing a wide variety of infections. Many *Enterococci* have plasmid-encoded resistance genes which cause less susceptible to several antimicrobial agents intrinsically including gentamicin and cefradine [31–33]. Biofilm formation has been identified as an essential factor in the evasion of the host’s immune response, the inhibitory or killing effects of antibiotics, and the pathogenesis of enterococcal infections [32, 34]. In addition, *Enterococci* are recognized as possessing a variety of virulence factors, which contribute to the mediation of adhesion, colonization, and invasion into the host tissues, modulation of the host immunity, and extra-cellular production of enzymes and toxins [35]. In this study, *Enterococcus* exhibited its intrinsic resistance to gentamicin and cefradine. Instead of being inhibited or killed, they proliferated in large quantities. A comparative genomic analysis discovered that the core-genome of *Enterococcus* obtains many genes related to carbohydrate metabolism and mannose, fructose, lactose, and galactose were the principal energy sources of *Enterococcus* [36]. Based on previous studies and the results presented here, we propose that the overgrown *Enterococcus* may cause colonic infection and homeostasis disorder by forming a biofilm, possessing virulence factors, and adjusting carbohydrate metabolism. The specific mechanism needs further verification.

Another noteworthy bacterium was *Clostridium*. As known to all, *C. difficile* and *C. perfringens* are high-risk infectious origins of AAD. The former can produce an enterotoxin (toxin A) and a cytotoxin (toxin B), which cause mucosal injury and colonic inflammation [7]. The later can produce potent protein toxins (α-toxin, β-toxin, ε-toxin, and ɩ-toxin), which cause many different histotoxic and enterotoxic diseases in humans and animals [37]. There was no direct taxonomic evidence for *C. difficile* and *C. perfringens* in our data, but a significantly increased abundance of *Clostridium* also attracted our attention. We blasted the original sequence pairs that were classified into the genus *Clostridium* to the NCBI database separately. Then, a suspected strain of *C. difficile* and a suspected strain of *C. perfringens* were found. However, the identity of the specific strain remains to be further confirmed.

## Conclusions

In summary, the bacterial diversity of the intestinal lumen was diminished after gentamicin and cefradine administration. The alterations in the abundance and composition of gut microbiota further led to the dysfunction of gut microbiota. More specifically, gentamicin and cefradine significantly increased the abundance of the opportunistic pathogens, of which *Enterococcus* and *Clostridium* are the most prominent and most worthy of attention.

## Methods

### Experiment animals

Six male and six female one-month-old specific pathogen free Kunming mice, weighing about 20 ± 2 g, were purchased from Hunan SJA Laboratory Animal Co., Ltd. (SCXK (Xiang) 2013–0004). These mice were housed under stable conditions (temperature 23–25 °C, relative humidity 50–70%, 12 h light/dark cycle) with unrestricted access to water and diet at the Experimental Animal Center of Hunan University of Chinese Medicine. The process of all animal experiments was conducted under animal protocols approved by the Institutional Animal Care and Use Committee of Hunan University of Chinese Medicine (Number: 20171202).

### Experiment reagents

Gentamycin Sulfate Injection (2 mL) and Cefradine Capsules (0.25 g) were obtained from Yichang Renfu Pharmaceutical Co., Ltd. (Batch No.: 5120106) and Shanxi C&Y Pharmaceutical Group Co., Ltd. (Batch No.: 110804). The mixture of antibiotics, at a concentration of 62.5 g·L^− 1^, was prepared by Gentamicin sulfate injection, cefradine capsules, and physiological saline, and then reserved at 4 °C.

### Animal experimental process and sample collection

After 2 days of adaptive feeding, twelve mice were randomly divided into the control group (ack, 3 male and 3 female) and the model group (am, 3 male and 3 female). Then, female mice and male mice in the same group were fed separately housing (three male or three female mice per cage). The model group mice were administrated with the antibiotics mixture 0.35 mL per time by gavage, twice a day for 5 days [3, 20]. Meanwhile, mice in the control group were given sterile saline with equal dose and frequency. The criteria of the AAD model are: ①Frequency of defecation increased (2–3 times per day); ②Feces becomes thin and soft; ③Anus becomes dirty. Twelve mice were sacrificed by cervical dislocation on the 8th day, and their intestinal contents in the colon were collected separately under sterile conditions. To control the difference induced by gender, the intestinal contents of one male and one female from the same group were mixed. Then, six samples were loaded into EP tubes separately and frozen at minus 80 °C.

### PCR amplification and sequencing

Metagenomic DNA was extracted from 6 samples using a modified cetyltrimethylammonium bromide (CTAB) method according to our previous reports [28, 38]. The concentration and purity were measured by an Ultraviolet Spectrophotometer. Then, according to the concentration test results, the integrity of the DNA sample was detected by 0.8% agarose gel electrophoresis. The metagenomic DNA was diluted and used as a template. Then, combined with bacterial 16S rDNA V4 region universal primer pair (520F: 5′-AYTGGGYDTAAAGNG-3′ and 802R: 5′-TACNVGGGTATCTAATCC-3′), the metagenomic DNA was amplified by PCR. After detected by 2% agarose gel electrophoresis, the PCR products were sequenced using the Illumina Miseq platform. PCR amplification and high-throughput sequencing were performed by Shanghai Personal Biotechnology Co., Ltd.

### Bioinformatics analysis

Raw pair-end sequences were further filtered using Qiime software (version 1.7.0, http://qiime.org/) and removed chimeras using Mothur software (version 1.31.2, http://www.mothur.org/) to obtain high-quality sequences which were finally used for subsequent analysis. Then, high-quality sequences were clustered into operational taxonomic units (OTUs) with 97% similarity (3% cutoff) using Qiime software, and chimeric sequences were identified and removed. Greengene database (Release 13.8 http://greengenes.secondgenome.com/) was used to assigning taxonomy to the OTU table by the blast method in Qiime platform. After quality-filtering, a modified OTUs table was used for further analyses. The Venn diagram between groups was generated using R software. Four common diversity indices were calculated using Mothur software and visualized using GraphPad software. Based on the information of abundance at the genus level, the community structure map was drawn using MetaPhaAn software. LEfSe analysis was implemented to find biomarkers between two groups using the online tool hosted on the Galaxy web application [19] (https://huttenhower.sph.harvard.edu/galaxy/). LEfSe determines the features (operational taxonomic units, or taxa) most likely to explain differences between classes by coupling standard tests for statistical significance with additional tests encoding biological consistency and effect relevance [19]. A file compatible with LEfSe was created using the table of relative abundance and uploaded to the web application on Galaxy to run the LEfSe analysis. *p*-value of < 0.05 was considered significant for statistical methods. Then, taxa with a logarithmic LDA score of over 4.0 were defined as biomarkers whose abundances were significantly increased compared to the other group.

### Statistical analysis

When appropriate, data were presented as mean & standard deviation ($\overline{x}$ ± SD). All statistical analyses were performed in R (version 4.0.2), and raw *p*-value < 0.05 and false discovery rate adjusted *p* (*p*FDR) < 0.05 were considered as statistically significant. Mann-Whitney U test (non-parametric) was utilized to evaluate differences in characteristics of the normal group and model group. *p*-values were adjusted by Benjamini & Hochberg (BH) correction.

## Data Availability

The datasets used and analyzed during the current study are available from the corresponding author on reasonable request.

## Abbreviations

AAD: Antibiotic-associated diarrhea
ADE: Adverse drug event
OTU: Operational taxonomic unit
CTAB: Cetyltrimethylammonium bromide
PCoA: Principal coordinates analysis
LDA: Linear discriminant analysis
LEfSe: Linear discriminant analysis effect size
F/B: Firmicutes to Bacteroidetes ratio
